# Regulatory T cells in the era of cell and gene therapy: biology, clinical translation, and future prospects

**DOI:** 10.3389/fimmu.2026.1762753

**Published:** 2026-03-27

**Authors:** Yohei Sato

**Affiliations:** 1 Core Research Facilities, Research Center for Medical Sciences, The Jikei University School of Medicine, Tokyo, Japan; 2 Laboratory of Immune Cell Therapy, Project Research Units, Research Center for Medical Sciences, The Jikei University School of Medicine, Tokyo, Japan; 3 Immunology and Allergy Research Unit, Department of Otorhinolaryngology, Head & Neck Surgery, Faculty of Medical Sciences, University of Fukui, Fukui, Japan

**Keywords:** clinical trial, good manufacturing practice (GMP), lentiviral (LV) vector, rapamycin, regulatory T cells (Treg)

## Abstract

Regulatory T cells (Tregs) play a central role in maintaining immune homeostasis. Therefore, owing to their unique immunomodulatory function, their adoptive transfer has been investigated as a novel therapeutic modality. Recent progress in cell manufacturing has allowed the application of Tregs as cell therapy products, and several are being tested in human clinical trials. Furthermore, several clinical trials are aimed at evaluating Treg modulation methods, including cytokine administration, monoclonal antibodies, Treg depletion, and adoptive Treg transfer. Among these, adoptive Treg transfer is a promising cell and gene therapy modality that is potentially beneficial for both genetic and non-genetic diseases, including autoimmunity. To determine the current trends in research on Treg-based therapies, we performed a database search and found that investigational clinical trials have been performed not only for autoimmunity but also for a wide range of diseases. In this mini review, we introduce early benchmarking Treg trials together with recent advances in research on Treg biology shown in non-genetic diseases and not just limited to autoimmunity.

## 1 Introduction

Since their discovery, regulatory T cells (Tregs) have been extensively studied in humans and mice and shown to play a central role in controlling immune responses (1). Tregs are characterized by their constitutive expression of forkhead box P3 (FOXP3) (2), the pivotal role of which has been demonstrated using genetic mutations in humans and mice. In particular, “scurfy mice” represent a model of lethal autoimmunity caused by Foxp3 deficiency (3). A mutation in human *FOXP3* results in IPEX (immune dysregulation, polyendocrinopathy, enteropathy, X-linked) syndrome, which is characterized by severe autoimmunity that begins in the neonatal period (4). Therefore, elucidating the biology of Tregs would contribute toward the therapeutic control of immune homeostasis in autoimmunity. The mechanisms of Treg-mediated suppression under physiological conditions are mainly explained by the production of inhibitory cytokines, local consumption of IL-2, inhibition of antigen-presenting cells, and ATP hydrolysis, depending on the environment and target cells (5). Under pathological conditions, a recent study indicated that Tregs may suppress immune reactions against cancer, particularly leukemia (6). Reducing or depleting Tregs in the tumor microenvironment may enhance the host immunity against tumors (7). Presently, Tregs are also considered key players in cancer immunotherapy (8). These close associations of Tregs with multiple disease mechanisms have led to an increase in clinical and translational studies of these T cells. Currently, Tregs are being used as a cell therapy modality either without or with gene engineering (e.g., viral gene transfer and CRISPR/Cas9 gene editing), which endows additional properties such as enhanced gene expression (9). In this review, the biology of Tregs and their clinical application in various diseases are discussed together with future perspectives on engineered Treg therapy.

## 2 Biology of Tregs

Tregs were initially identified as CD4^+^CD25^+^ cells that contribute to immune tolerance in mice (1). FOXP3, the master transcription factor identified in Tregs, was shown to mediate immune suppressive function through the upregulation of inhibitory molecules such as cytotoxic T-lymphocyte-associated protein 4 (CTLA-4), interleukin-2 (IL-2) receptor subunit alpha (CD25), and glycoprotein A repetitions predominant (GARP) (2). Subsequently, Tregs were found to express interleukin-7 receptor subunit alpha (CD127) at a low level and were thus re-defined as CD4^+^CD25^+^CD127^low^FOXP3^+^ T cells (10). The discovery regarding CD127 enabled the flow cytometric sorting of FOXP3^+^ Tregs without the need for FOXP3 staining, the latter of which requires tissue fixation and permeabilization. Moreover, IPEX syndrome was shown to be caused by genetic mutations in the *FOXP3* locus that resemble the *Foxp3* gene mutation in scurfy mice, further confirming the importance of this transcription factor (11, 12). These clinical and experimental observations have enhanced our understanding of the clinical and molecular aspects of Tregs. Furthermore, the potential association of Tregs with tumor immunity and cancer progression has driven scientists to study these T cells in the tumor microenvironment or tumor tissues (13). Recent advances in immune checkpoint blockade, including anti-programmed cell death protein 1 (PD-1), anti-programmed death-ligand 1 (PD-L1), and anti-CTLA-4 immunotherapy, also support the importance of local immune suppression by tumors that utilize FOXP3^+^ Tregs to escape tumor immunity (14). Therefore, Tregs in autoimmunity, which is caused by a lack of tolerance to self-antigens, have been studied in the context of cancer/tumor immune escape (15). These two approaches also indicate the importance of appropriate maintenance of the immune system according to the local immune context.

## 3 Treg clinical trials using polyclonally expanded Tregs

Unmodified, polyclonally expanded Tregs have been investigated in various clinical trials. For instance, polyclonally expanded Tregs have been studied in xenogeneic GvHD and type 1 diabetes (T1D) in early studies (16–19). The safety and feasibility of adoptive Treg transfer have been confirmed across multiple clinical trials. While the safety and tolerability of polyclonal Treg transfer have been confirmed, the therapeutic efficacy of Tregs remains heterogeneous. Compared to xenogeneic GvHD and T1D, organ transplant recipients showed more promising clinical efficacy, which may be explained by early intervention and the preventative role of Treg transfer (5). In particular, with regards to liver transplantation, polyclonal Treg transfer facilitated the discontinuation of immunosuppressive agents (20). Moreover, in living-donor kidney transplantation, polyclonal Treg transfer reduced infection rates, while the rejection rate was not significantly reduced (21). Collectively, the therapeutic efficacy of unmodified Tregs has been inconsistent, due to heterogeneous disease targets and variability in cell production and clinical protocols. Notably, safety and tolerability have been consistently confirmed among various studies, supporting further investigation.

## 4 Current Treg clinical trials

**Figure 1:**
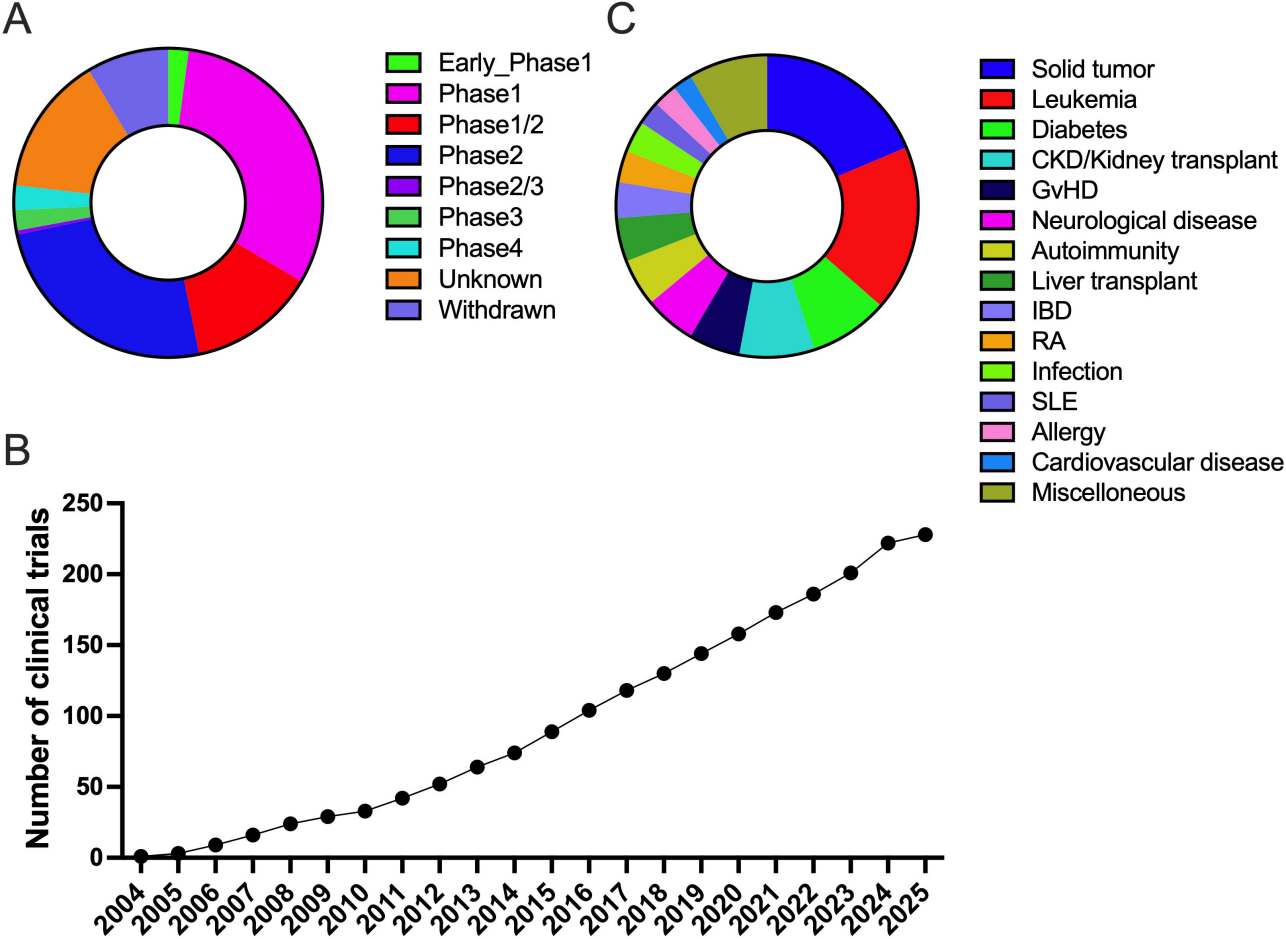
Current status of interventional clinical trials related regulatory T-cell. **(A)** Phases of clinical trials related to Regulatory T cells. **(B)** The cumulative number of clinical trials related to Regulatory T cells. **(C)** The indications of clinical trials related to Regulatory T-cell.

Because of their useful biological properties, Tregs have been investigated in various clinical trials. To better understand the overall demographics of the various cohorts, interventional clinical trials registered at ClinicalTrials. gov (http://clinicaltrials.gov) were analyzed and those involving “regulatory T cells” were extracted from the database in March 2025. Over 200 active clinical trials ranging from phases 1 to 4 have been registered. Indeed, most clinical studies were in the early phase (phase 1 or 2), and few were in the late phase (phase 3 or 4) (**Figure 1A**). Furthermore, the number of investigational clinical trials on Tregs has increased over time (**Figure 1B**). Based on the currently ongoing early-phase trials, a future increase in late-phase trials is indicated.

As pointed out in another similar literature review, the most common targets are cancer, autoimmunity (T1D, graft-versus-host disease, inflammatory bowel disease, rheumatoid arthritis, and systemic lupus erythematosus), hematological malignancies, and solid organ transplantation (**Figure 1C**) (22). Recently, neurological disorders (multiple sclerosis, Alzheimer’s disease, and amyotrophic lateral sclerosis), infectious diseases (COVID-19), allergies (asthma and allergic rhinitis), and cardiovascular diseases (coronary artery disease and heart failure) were also targeted in the clinical trials. Although the association of Tregs with neurological diseases has been reported, novel immunosuppressive strategies (e.g., adoptive transfer or Treg enhancement) for autoimmune neurological diseases have yet to be developed (23). Moreover, allergy is also driven by the Th2 immune response, and the association of Tregs with allergic diseases is known (24). Therefore, the use of Tregs for allergic conditions is not surprising since these T cells are considered major drivers of allergen-specific immunotherapy (25). Importantly, the only clinical trial aimed at a genetic immune disease is that of CD4LVFOXP3 for IPEX syndrome (26). Regardless of the rarity of the disease, the biology and manufacturing of genetically engineered Tregs would be beneficial for both genetic autoimmune disorders and other non-genetic conditions (27). These data suggest the possible application of adoptive Treg transfer and Treg-targeted therapy for non-immunological diseases that could benefit from modulation of these T cells.

**Figure 2:**
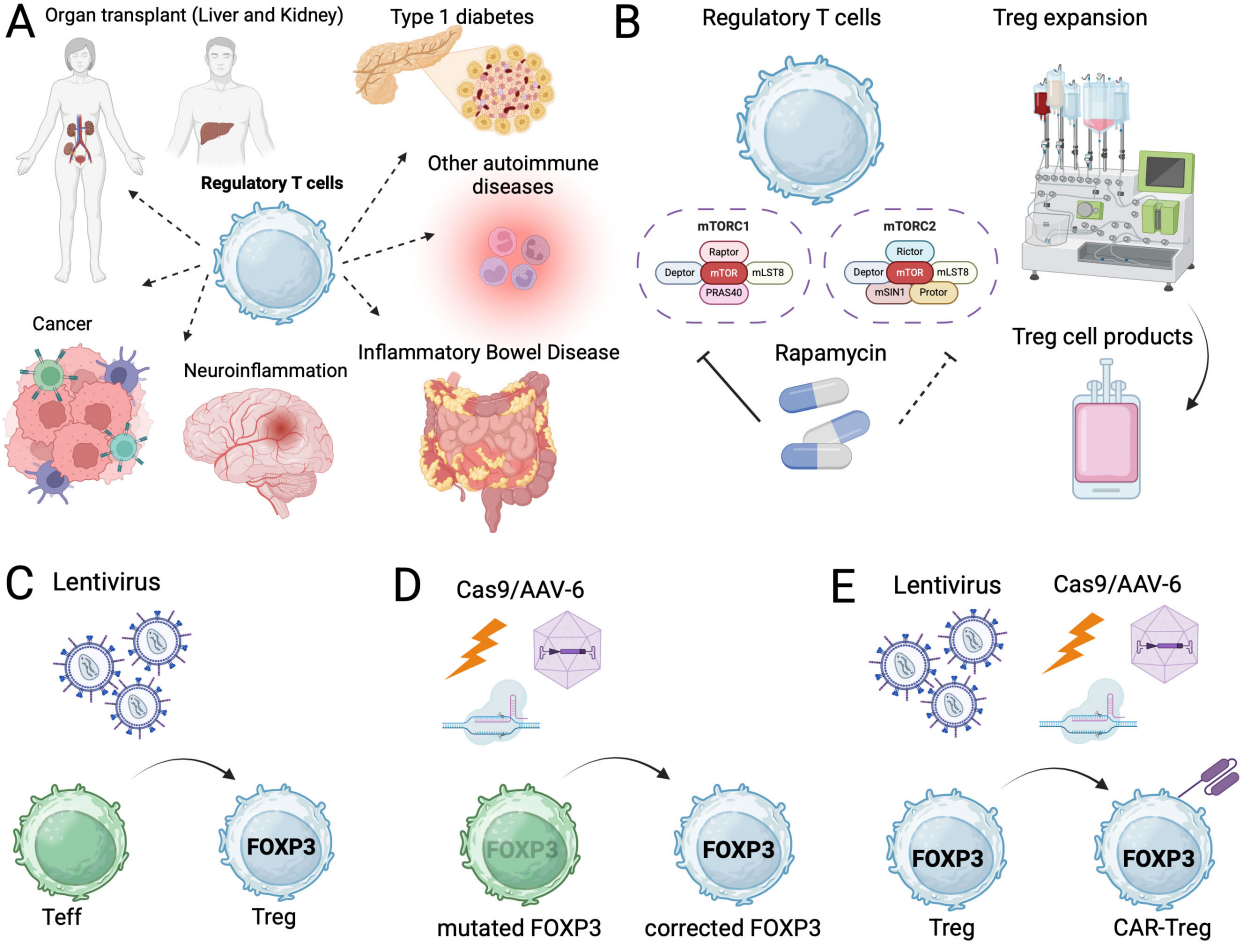
Regulatory T-cell therapy for immune-mediated diseases. **(A)** The indications of Regulatory T-cell therapy. **(B)** The role of rapamycin on selective expansion of the Tregs and example of Treg manufacturing using rapamycin in the clinical trials. **(C)** Engineered regulatory T-cell therapy using lentiviral FOXP3 gene transfer. **(D)** CRISPR/Cas9 mediated FOXP3 gene editing. **(E)** CAR-Treg generation therapy using lentiviral gene transfer or CRISPR/Cas9 mediated gene editing. Figure was created with (https://BioRender.com/s3gx5vw).

## 5 Non-genetic engineering of Tregs

Recent progress in cell manufacturing has allowed the application of Tregs as cell therapy products, and several are being tested in human clinical trials. It is possible to isolate Tregs with a certain degree of purity and expand them using *in vitro* large-scale conditions compatible with good manufacturing practice (GMP)-grade manufacturing (28). The use of *ex vivo*-expanded Tregs for type 1 diabetes (T1D) was tested in an early benchmark study, which successfully demonstrated the safety of the cell product (18). Since then, several clinical trials on the use of expanded Tregs to suppress autoimmunity have been conducted (**Figure 2A**).

Rapamycin preferentially inhibits mTORC1, while Tregs require tightly regulated mTOR signaling, with basal mTORC1 activity being essential for their survival and function, whereas mTORC2 is dispensable for survival but contributes to lineage stability. While conventional T cells heavily rely on mTORC1-dependent glycolysis, Tregs can utilize oxidative phosphorylation for survival. Therefore, the addition of rapamycin, which preferentially inhibits mTORC1, may contribute to the selective expansion of Tregs (29, 30). The expansion of Tregs with their suppressive function preserved has proven to be difficult owing to the plasticity of the T cells. Therefore, the rapamycin-mediated metabolic engineering of Tregs allows them to be expanded without any gene modifications (**Figure 2B**). In addition to the selective expansion, rapamycin stabilized TSDR demethylation and improved mitochondrial fitness (31, 32). Recently, the safety of rapamycin-promoted *ex vivo*-expanded Tregs was demonstrated in a large study on living kidney donor transplantation (21). Rapamycin, which preferentially expands Tregs, allowed the cells to maintain their expression of FOXP3 and immune suppressive functions. However, the continuous provision of rapamycin may not be possible, especially after adoptive transfer. Interestingly, in that same study, the incidence of infections was significantly reduced by Treg infusion, and the safety of the strategy was guaranteed. Nonetheless, follow-up studies to validate the functions of metabolically engineered Tregs are needed. Recently, rapamycin was shown to stabilize Tregs in both patients with neurological diseases and those with IPEX syndrome (33, 34).

Similar to rapamycin, low-dose IL-2, which may stimulate Tregs, has been investigated in clinical trials; however, the results do not support its use for the *in vivo* expansion of Tregs (35). On the other hand, low-dose IL-2 has shown clinical efficacy in patients with systemic lupus erythematosus (SLE) by promoting Treg tissue homing (36). However, off-target activation of other immune cells and a limited *in vivo* half-life make it challenging to achieve long-term therapeutic efficacy with IL-2, compared to Treg adoptive transfer, which may survive long term *in vivo*. In addition to wild-type IL-2, orthogonal IL-2 has been investigated to achieve precise tuning of Treg activity and to avoid off-target effects (37). Moreover, orthogonal IL-2 selectively expands Tregs in xenogeneic GvHD mouse models (38). Therefore, these pharmacological approaches may enhance the efficacy of polyclonally expanded Tregs and represents a promising therapeutic strategy for acquired autoimmune diseases.

## 6 Genetic engineering of Tregs

Since the discovery of *FOXP3*, multiple research groups have attempted to transduce the gene into human effector T-cells (39, 40). Constitutive FOXP3 expression in those cells results in a Treg-like phenotype and immune suppressive functions (**Figure 2C**). Together with successful lentiviral-based gene therapy products (including CAR-T cells), *FOXP3*-transduced CD4^+^ T cells can be generated under GMP-grade conditions (41, 42). Clinical trials using effector T cells carrying lentiviral-transduced *FOXP3* (CD4LVFOXP3) are currently ongoing (26). In addition to viral gene transfer, CRISPR/Cas9-mediated *FOXP3* correction or stabilization also allows for the generation of Treg-like cell products (43, 44). Unlike with viral transduction, targeted gene integration is possible in gene edited Treg-like cells, thereby avoiding off-target effects (**Figure 2D**). However, the induction of high and stable FOXP3 expression may not be possible with gene editing compared with viral-mediated gene transfer due to the physiological regulation of gene expression (27). Both approaches have advantages and disadvantages, and future studies to achieve cell- and gene-therapy-mediated immune modulation are warranted. An alternative approach is genetically modified hematopoietic stem cell therapy. As FOXP3 can be harmful to hematopoietic stem cells, a synthetic promoter that drives *FOXP3* expression during T-cell development can rescue the phenotype of scurfy mice (45).

Another genetic engineering approach for immune regulation is the use of “CAR-Tregs,” which are generated similarly to conventional CAR-T cells (46). CAR-Tregs are generated from Tregs, whereas CAR-T cells are derived from effector T cells (**Figure 2E**). Because of the similarities in their manufacturing processes, CAR-Tregs have also been investigated in a clinical trial (47). These genetically engineered Treg-like cell products have the potential to suppress immune reactions more efficiently than expanded Tregs without gene modifications can do (27). Furthermore, CAR-Tregs should show antigen specificity, which may result in their higher potency and a lower risk of adverse events (48). While early studies targeted alloantigens (e.g., HLA-A2), a broader range of molecules has since been targeted, expanding the spectrum of disease indications. Similar to polyclonally expanded Tregs, CAR-Tregs have been investigated for treating autoimmune diseases including type 1 diabetes and inflammatory bowel disease (IBD) (49, 50). Notably, strong CAR-mediated signaling may induce inflammatory gene expression in CAR-Tregs (51). Although CAR-Tregs undoubtedly exhibit higher antigen specificity compared to polyclonally expanded Tregs, their unique biological properties require further molecular and functional investigation to enable efficient and safe clinical translation.

## 7 Balance between effectiveness and safety

A protocol for the isolation and expansion of Tregs without genetic engineering has been established, and various technical modifications are still being explored (52). From a clinical or scientific perspective, the quality and quantity of Treg products can be determined on the basis of other Treg products. Moreover, as long as the quality of the Treg products is similar, potential toxicity and adverse events during clinical trials are to be expected (53). However, important consideration must be given to the effects of the patient status, as the product can be altered by medical conditions such as autoimmunity, cancer, organ transplantation, and surgical procedures. Therefore, Treg products must be tested in preclinical models that resemble the patient conditions for whom the Treg product is being developed (54).

However, gene modification is a variable that could potentially influence the quality and potency of Treg products both positively and negatively (55). Recently, CAR-Tregs targeting the OX40 ligand (OX40L) were shown to effectively control immune reactions in mice (56). Interestingly, OX40L-targeted CAR-Tregs controlled alloreactive responses more efficiently than control (mock transduced) Tregs did. Since a cell population similar to that of genetically modified Tregs may not exist in the normal immune system, careful investigation is necessary before such types of promising T-cell products can be brought to clinical trials (57). Therefore, the further study of highly potent genetically engineered Tregs for treating genetic and non-genetic autoimmune disorders and various medical conditions would be immensely beneficial. Notably, Tregs play multiple roles in the immune system (especially in immune modulation) and tissue repair and may be relevant to the aging process.

## 8 Limitations and challenges in regulatory T-cell therapy

Despite substantial progress, several biological and technical constraints continue to limit the consistent clinical efficacy and late-phase translation of regulatory T-cell (Treg)–based therapies (58). These challenges span antigen specificity, lineage stability, manufacturing standardization, safety of engineered products, and the lack of predictive biomarkers (59). A critical appraisal of these issues is essential to guide next-generation strategies.

Polyclonally expanded Tregs offer logistical advantages because they can be generated without prior knowledge of the target antigen and have an established safety record in early-phase trials (18). However, their therapeutic efficacy is often modest, largely due to limited accumulation at disease sites and the need for high cell doses, which increases the risk of systemic immunosuppression (60). In contrast, antigen-specific Tregs, including TCR-engineered and chimeric antigen receptor (CAR)–Tregs, provide enhanced potency and localized immune regulation at lower doses (46). Nevertheless, their clinical application is constrained by incomplete knowledge of disease-relevant antigens, antigen heterogeneity, and epitope spreading in complex immune-mediated disorders (61). Furthermore, increased manufacturing complexity and the potential for excessive receptor signaling introduce additional translational barriers.

The stability of the FOXP3-dependent transcriptional and epigenetic program remains a central determinant of therapeutic efficacy (39). Pro-inflammatory cytokines, strong co-stimulatory signals, and metabolically unfavorable tissue environments can impair suppressive function and promote the acquisition of effector-like phenotypes (62). Importantly, the phenotype of ex vivo–expanded Tregs does not necessarily predict their functional state after infusion, as local cues within inflamed tissues can reshape cellular identity (63). This context dependency represents a major obstacle to achieving durable and reproducible clinical responses.

Current good manufacturing practice (GMP)–compliant production of Treg cell products is associated with substantial variability in yield, purity, and functional potency (64). Differences in starting material, particularly in patients with advanced disease or chronic inflammation, directly affect expansion capacity and lineage stability (40). The absence of a unique surface marker for Tregs complicates purification, and contaminating conventional T cells may preferentially expand during culture. In addition, prolonged culture periods, high production costs, complex quality-control requirements, and the lack of standardized potency assays limit scalability and inter-trial comparability (65). These factors collectively hinder the transition from early-phase to large multicenter studies.

Genetic engineering strategies aim to enhance specificity, stability, and persistence but introduce new safety considerations (66). Genome editing carries the risk of off-target modifications and unintended genomic alterations, necessitating comprehensive molecular quality control. Viral vector–based approaches may lead to variable transgene expression and insertional mutagenesis, although the latter risk has been reduced with modern vector design. CAR-Tregs present additional challenges, as tonic or excessive CAR signaling can destabilize the regulatory phenotype and induce inflammatory transcriptional programs (67). Moreover, the distribution of target antigens in non-diseased tissues raises the possibility of off-target immunosuppression, particularly in long-lived cell products.

A major bottleneck in the field is the absence of validated biomarkers and potency assays that reliably predict *in vivo* efficacy (68). The biodistribution, tissue residency, and long-term persistence of infused Tregs remain incompletely understood, as does the optimal dosing strategy and the need for combination therapies that support survival and function (69). In addition, patient stratification criteria are poorly defined, and the mechanisms underlying therapeutic non-response are largely unknown. Long-term safety data, including the effects on host defense, tumor surveillance, and vaccine responses, are still limited (41). Addressing these gaps will be critical for the design of next-generation trials and for the successful integration of Treg therapy into routine clinical practice.

Collectively, these limitations highlight that the clinical translation of Treg therapy is not solely a matter of improving cell manufacturing but requires a deeper understanding of tissue-specific immune regulation, context-dependent stability, and mechanism-based patient selection. Future advances will likely depend on integrated approaches combining antigen specificity, molecular stabilization, controlled *in vivo* support, and robust predictive biomarkers.

## 9 Future perspectives of engineered regulatory T-cell therapy

Since their original identification, Tregs have been studied in a variety of contexts not limited to autoimmunity, such as in cancer, allergies, infections, and cardiovascular diseases. Owing to their pivotal roles in immune homeostasis and regulation, Tregs have the potential to be utilized for the treatment of various diseases, thus leading to an increase in clinical and translational studies of these T cells (70). Additionally, their functions are not limited to immune suppression, and they can enhance tissue repair and regeneration (71). Indeed, Tregs accumulated in damaged skin have been shown to enhance wound healing, and their local administration improves scar healing (72, 73). Recently, Tregs were also demonstrated to support angiogenesis and stem cell maintenance (74). Moreover, because the aging process also affects the function of Tregs, their association with senescence needs to be studied in greater detail (75).

In addition to their unique biological properties, recent advances in molecular technologies have enabled the genetic engineering of Tregs. In CAR-T and CAR-Treg production, CRISPR/Cas9 has become a major genome-editing strategy. Base editing and prime editing may offer more precise and safer integration of gene-expression cassettes. Moreover, the addition of pro-regulatory cytokines and deletion of inflammatory programs may stabilize engineered Tregs, depending on the target disease. Recently, local immune environments have been extensively studied in various diseases, including cancer and autoimmunity, revealing factors that may alter the stability and function of resident immune cells. Improved immunological understanding may guide molecular and cytokine engineering strategies for more effective immune regulation.

## 10 Conclusion

Regulatory T cells have emerged as versatile therapeutic tools with the capacity to restore immune homeostasis, promote tissue repair, and modulate disease-specific immune responses across diverse clinical settings. Advances in cell manufacturing, metabolic modulation, and precise genetic engineering—particularly CAR and genome-editing technologies—are steadily improving their stability, specificity, and functional potency while highlighting the need for careful evaluation of safety and context-dependent efficacy. Continued integration of immunological insights with bioengineering strategies will be essential to translate engineered Treg therapy into broadly effective and durable clinical applications.
